# Relative importance of climatic, geographic and socio-economic determinants of malaria in Malawi

**DOI:** 10.1186/1475-2875-12-416

**Published:** 2013-11-14

**Authors:** Rachel Lowe, James Chirombo, Adrian M Tompkins

**Affiliations:** 1 Abdus Salam International Centre for Theoretical Physics, Trieste, Italy; 2 Institut Català de Ciències del Clima (IC3), Barcelona, Spain; 3 Ministry of Health, Lilongwe, Malawi

**Keywords:** Malaria, Climate, Socio-economic, Statistical model, Confounding factors, Random effects, Spatial correlation

## Abstract

**Background:**

Malaria transmission is influenced by variations in meteorological conditions, which impact the biology of the parasite and its vector, but also socio-economic conditions, such as levels of urbanization, poverty and education, which impact human vulnerability and vector habitat. The many potential drivers of malaria, both extrinsic, such as climate, and intrinsic, such as population immunity are often difficult to disentangle. This presents a challenge for the modelling of malaria risk in space and time.

**Methods:**

A statistical mixed model framework is proposed to model malaria risk at the district level in Malawi, using an age-stratified spatio-temporal dataset of malaria cases from July 2004 to June 2011. Several climatic, geographic and socio-economic factors thought to influence malaria incidence were tested in an exploratory model. In order to account for the unobserved confounding factors that influence malaria, which are not accounted for using measured covariates, a generalized linear mixed model was adopted, which included structured and unstructured spatial and temporal random effects. A hierarchical Bayesian framework using Markov chain Monte Carlo simulation was used for model fitting and prediction.

**Results:**

Using a stepwise model selection procedure, several explanatory variables were identified to have significant associations with malaria including climatic, cartographic and socio-economic data. Once intervention variations, unobserved confounding factors and spatial correlation were considered in a Bayesian framework, a final model emerged with statistically significant predictor variables limited to average precipitation (quadratic relation) and average temperature during the three months previous to the month of interest.

**Conclusions:**

When modelling malaria risk in Malawi it is important to account for spatial and temporal heterogeneity and correlation between districts. Once observed and unobserved confounding factors are allowed for, precipitation and temperature in the months prior to the malaria season of interest are found to significantly determine spatial and temporal variations of malaria incidence. Climate information was found to improve the estimation of malaria relative risk in 41% of the districts in Malawi, particularly at higher altitudes where transmission is irregular. This highlights the potential value of climate-driven seasonal malaria forecasts.

## Background

Malaria is one of the greatest public health problems in Malawi, placing children under the age of five and pregnant women at the highest risk of the disease. It is estimated that about 6 million clinical cases of malaria (for both under and over five years age groups) are reported annually [1], in a population of approximately 15 million [2]. *Plasmodium falciparum* is the leading malaria-causing parasite in Malawi and accounts for most of the cases reported. The 2010 Malaria indicator survey reported prevalence of 43.3% [3]. In a bid to reduce the spread of the disease, the Malawi government has embarked on different interventions such as vector control through indoor residual spraying and insecticide-treated nets (ITN) [4]. The latter primarily focuses on the high-risk groups of pregnant women and children under the age of five and is the main intervention method in Malawi. The government is now encouraging the use of long-lasting insecticidal nets of which there are mass distributions conducted periodically. Several Non Governmental Organizations (NGOs) and international organizations such as the World Health Organization (WHO), the President’s Malaria Initiative and the Global Fund are involved in malaria intervention activities. However, these NGOs usually work in selected districts and as such, some districts receive no aid. Despite the scaling up of malaria control measures over the last decade, the recent data have not suggested a decrease in the burden of disease [4,5].

Another challenge that the government faces in the fight against malaria is the detection of the disease at the health facilities, especially those in rural areas without the necessary laboratory facilities for testing. Presumptive malaria diagnosis has been used in the past and this led to over-diagnosis of malaria cases and consequently poor guidance to map regional risk and manage the disease. In 2011 the government of Malawi adopted the WHO recommendation that tests should be carried out in all suspected malaria cases and rapid diagnostic tests (RDT) were subsequently progressively introduced to health facilities across the country [6].

The focus of the government through the National Malaria Control Programme is to achieve universal coverage in the prevention and treatment of malaria. The aim is that by the year 2015, the 2010 levels of malaria morbidity and mortality will be reduced by half [1]. In order to achieve this goal, there is a pressing need for tools that can be implemented rapidly and cost-effectively to mitigate the burden of malaria. The government needs to know which districts are most at risk and when. Defining the spatial distribution of a disease within a country or region allows public health decision makers to identify zones susceptible to epidemics and to target resources toward those areas at greatest risk [7].

Climate variability can affect malaria transmission, both in terms of spatial and seasonal distribution, inter-annual variability and epidemic potential [8]. Rainfall can affect the availability of mosquito breeding, developmental and resting sites [9], while temperature influences the rate of development of immature stages and adult survival rate, biting frequency and the extrinsic incubation periods (the period between infection of the vector and the vector’s ability to infect the next susceptible host) of disease agents [10]. Climate variability and change in the epidemiology of mosquito-borne diseases are complex. While increasing temperature speeds up vector, larvae and parasite biological cycles, high temperatures can increase mosquito and larva mortality [11,12] and prevent malaria transmission above an upper temperature limit in the range of 33-39°C [13]. Likewise, rainfall can promote transmission by creating ground pools and other breeding sites, but heavy rains can have a flushing effect, cleansing such sites of early stage larvae [14,15]. The relationships between malaria and climate have been well documented and statistical and dynamical models have been developed to represent them. With such models, climate observations and forecasts could be used to predict epidemics on monthly to seasonal time scales [16-23]. In addition to the climatic factors that affect malaria transmission, consideration of the interaction of ecological variables with human behaviour and the urban environment is also important. Predictive models for malaria morbidity risk should also include potentially important non-climate variables that can affect population vulnerability and alter the underlying spatial distribution of infectious disease hazard associated with climate and environment [24].

The socio-economic status of a population can lead to conditions and environments conducive to vector proliferation and enhanced disease transmission. For example, rapid urbanization in Africa has contributed to unprotected water reservoirs, poor housing and lack of sanitation, which have implications for malaria transmission and epidemiology [25]. Poor sanitation in a household can lead to stagnant water holes that can act as breeding sites. Similarly, poor water storage and sanitation can provide breeding sites for mosquitoes around the household. A previous study of malaria risk in Ethiopia found that households with no toilet facilities were more likely to test positive for malaria [26]. That said, it is believed that those living in urban areas in Malawi are at a reduced risk of malaria compared to their rural counterparts. The 2010 Malawi Demographic and Health Survey reported that 30.7% of those surveyed in urban areas had fever in the preceding two weeks before the survey as compared to 35.1% in rural areas [27]. Kelly-Hope and McKenzie [28] report lower transmission intensities in urban African environments relative to rural areas. Urban areas possess qualities that reduce population vulnerability, compared to their rural counterparts. For example, literacy levels are generally higher and access to prevention interventions is greater than in rural settings. A study in Malawi found that the poorest populations in rural areas are not reached by intervention methods and ITN ownership was associated with living in urban areas and higher educational levels [29]. Further explanations posed as to why malaria transmission is lower in urban areas include pollution, which affects larval habitats and their life cycles [25,30], mosquito avoidance behaviour by urban dwellers [25] and higher population densities resulting in lower biting rates [25,31]. In rural locations, one room households are likely to be associated with greater risk of malaria compared to houses with more rooms, since such homes are unlikely to have sufficient nets for all members of extended families that frequently occupy such dwellings in rural Malawi. A study in Kenya found that adult mosquito abundance was significantly associated with traditional housing, with more mosquitoes found in grass thatched and mud wall houses [32].

Education status is a socio-economic factor that may impact malaria prevalence. Education status tends to affect the knowledge about malaria prevention and control among the population. Over recent years there has been emphasis on the idea that improving knowledge about malaria in communities will lead to better use of interventions [33,34]. A study in India found educated respondents were more knowledgeable about malaria than the illiterate [35]. Literacy has been found to be positively associated with parasitaemia in Kenya [36]. Nevertheless, some of the observed association between disease prevalence and education may also be due to its role as a proxy for poverty.

Concerning interventions, malaria prevalence is expected to reduce as ITN distribution rates increase. The National Malaria Control Programme has been distributing ITNs freely with the purpose of reducing prevalence amongst the most vulnerable groups (children and pregnant women). Prevalence is expected to drop in areas with a high distribution rate, given that this results in greater usage. However, in Malawi, where the use of mosquito nets is the most widely used intervention, owing to its low cost, net usage is still not as high as anticipated, despite several mass distribution campaigns.

Although climate demonstratively defines the temporal limits of the transmission season and spatially demarks endemic, epidemic and malaria free zones, the role that climate variations play in governing year to year variability in malaria morbidity rates is not well understood. Previous modelling efforts in Malawi used spatial analysis to predict and map malaria risk across the country, by modelling point-referenced prevalence of infection data, with topographical and climatic factors as explanatory covariates [37]. Kazembe [38] then went on to profile the spatial variation of malaria risk in under fives, using Bayesian spatial analysis to investigate the possible association of disease risk with environmental factors at the sub-district level in northern Malawi.

This study extends that of Kazembe [38] by using a spatio-temporal dataset stratified by age (under five years and five years and over) for the whole country, spanning several years (July 2004 - June 2011). This improves the ability of the model to inform malaria risk trends in Malawi as a whole. Further, clinical malaria data is used, which, although has the propensity to over-estimate the risk, provides a general picture of the overall malaria burden and resources required to decrease malaria risk. As well as environmental data, socio-economic determinants are also considered to better account for spatial variations in malaria risk. The aim of this analysis is to investigate the spatial and inter-annual variations in malaria morbidity in Malawi and to determine how much, if any, of the inter-annual variability is due to climate variability relative to other non-climatic factors. This is accomplished by using a Bayesian statistical modelling approach, which is increasingly being used for mapping and predicting the risk of infectious diseases and can account for spatial dependence and unknown random effects [38-44]. Here, the spatio-temporal model framework developed by Lowe [41,43] is applied and extended to analyze a newly compiled database of malaria morbidity in Malawi and determine which socio-economic, geographic and climate effects explain the spatial and inter-annual variability.

## Methods

### Study area

Malawi is a small country in Southern Africa, bordered by Tanzania to the north, Zambia to the west and Mozambique to the south, with an area of about 120,000 km^2^. Malawi is divided into 28 districts within three administrative regions. The country has a varied topographical landscape with highland areas in the northern districts of Chitipa and Rumphi, where the Nyika Plateau is located. High mountainous areas also include Dedza, the Zomba Plateau and Mulanje Mountain in the south east corner of Malawi. These areas have lower temperatures, which reduce the transmission of the disease. In contrast, low-lying areas are found in the Shire river valley and along Lake Malawi. Districts in these areas experience higher temperatures and generally report higher malaria incidence. Lilongwe and Kasungu plains in the central region are the two biggest plains in the country.

Malawi falls within the tropics between latitudes 9°S and 18°S and longitudes 32°E and 36°E and experiences two distinct rainy and dry seasons. Malaria transmission mainly occurs between November and April during and shortly after the warm and wet months in which 95% of the annual precipitation is recorded. Average temperatures vary between 25-37°C in this period [45], which provides the best environmental conditions for the breeding of the malaria vectors. There is a considerable drop in malaria cases during the dry season between May and October, with mean temperatures varying between 17-27°C.

The majority of the population of Malawi live in rural areas and are involved in small scale subsistence farming. The rural population typically live in traditional dwellings with mud walls and a thatched roof. Overall, illiteracy is still a challenge in Malawi with an adult literacy rate in 2009 of 70% [46].

### Data sources

#### Malaria data

Malaria case information was obtained from the Health Management Information Systems (HMIS) operated by the Ministry of Health (MOH), a database which records routine health data. In this system, both clinically and non-clinically diagnosed malaria cases disaggregated by age are routinely collected from all health facilities across Malawi, aggregated monthly to the district level. The HMIS in its current form was developed in 2002 but only started functioning nationally in 2004. During the first two years of being established, there were many challenges as the system did not function well in most districts. As RDT testing was introduced in 2011, the majority of diagnosed cases are non-clinically diagnosed suspected cases, which is likely to cause a significant overestimation of cases. From the district offices, data is sent to the national central database at the Ministry of Health every quarter. The malaria data used for this study was stratified by age (under five years and five years and over) for the period July 2004 - June 2011, as the reporting year starts in July and ends in June. Population and other demographic indicators were obtained from the National Statistical Office (NSO). The population figures for the districts were obtained from the population projections report by the NSO based on the 1998 population and housing census [47].

#### Socio-economic data

A database of potential drivers of malaria risk in Malawi was collated for every district for each month between July 2004 - June 2011 (see Table 1). Certain variables were calculated using population estimates, for example population density, the proportion of the population living in urban areas and the proportion of health facilities and ITN distribution per inhabitant. Data on urbanization, housing, health care provision, sanitation and literacy levels were obtained from Welfare Monitoring Surveys (WMS) [46] conducted annually by the NSO (see Table 1).

**Table 1:** Source and original resolution of datasets

**Data**	**Description**	**Spatial resolution**	**Temporal resolution**	**Source**
Malaria cases	Malaria cases from July 2004 - June 2011 reported at health facilities	District	Monthly	HMIS, Ministry of Health
Area	Land area of the districts in Malawi	District		Unpublished reports
Population	Population projections based on the 1998 population and housing census.	District	Yearly	NSO population projections [47]
Urban population	Population residing in the urban centres of Malawi	District	Yearly	NSO population projections [47]
One room	Proportion of dwelling units with one sleeping room	District		Demographic and Health Survey [27]
No toilet facilities	Percentage of households without toilet facilities	District		Welfare Monitoring Survey [46]
Literacy rate	Proportion of those aged five and above who can read and write in any language	District		Welfare Monitoring Survey [46]
No school	Proportion of the adult population who never attended school	District		Welfare Monitoring Survey [46]
Traditional housing	Defined as a dwelling with mud walls and a thatched roof	District		Welfare Monitoring Survey [46]
ITN distribution	Number of nets distributed by government, NGOs and some international agencies	District	Yearly	Unpublished reports by the National Malaria Control Programme
Number of health facilities	Network of health facilities operated by government and religious bodies	District		MOH database
Precipitation	Precipitation estimates (units: mm day^-1^)	10km grid	Daily	FEWS CPC/Famine Early WarningSystem Daily Rainfall Estimates [48]
Temperature	Temperature reanalysis data (units: °C)	80km grid	Daily	ERA-Interim reanalysis [49]
Altitude	Digital elevation data	90m grid		Shuttle Radar Topography Mission 90 m dataset [50]

#### Climatic and geographic data

Monthly precipitation estimates (units: mm day^-1^) were derived from the CPC/Famine Early Warning System Daily Rainfall Estimates (RFE 2.0) over Africa, available at a 10km resolution [48]. Temperature estimates (units: °C) were derived from the ERA-Interim reanalysis produced by the European Centre for Medium-Range Weather Forecasts (ECMWF) [49], with a coarser resolution of 80km. Altitude data was obtained from the Shuttle Radar Topography Mission 90m digital elevation dataset [50]. Climatic and topographic data were calculated for each district in Malawi using an interpolation method to relate gridded products to administrative districts [51]. Districts were further grouped into administrative regions (north, central, south) and ecological zones (lakeshore, lowland, highland, highland/lakeshore, highland/lowland).

### Statistical analysis

The objective of statistical modelling is to determine a minimal adequate model from the large set of potential models that might be used to describe the given set of data. Selecting few predictors from among a large number of potential candidates is a major challenge and can easily become arbitrary. An explanatory variable should only be included in the model if it significantly improves the fit of the model. A limitation of standard statistical modelling approaches is that they assume independence between survey locations and neglect potential spatial dependency between neighbouring locations due to unobserved common exposures [44]. Estimation of standard errors of explanatory variables is biased if overdispersion or spatial correlation is not taken into account within a model. Geostatistical models take into account spatial correlation by incorporating additional location-specific random effect parameters into a model. Such correlations may arise from factors such as variations in health system performance, intervention coverage or population immunity. Bayesian geostatistical approaches are increasingly used for mapping and predicting the risk of infectious diseases [52]. Ideally every possible combination of variables would be tested and compared in a Bayesian framework. However, this is not presently a viable approach as it is extremely time and computing-intensive. The most practical approach is to reduce the list of potential explanatory variables using general regression selection methods, before moving to a Bayesian context [40].

In this paper, the extent to which spatio-temporal variations in malaria risk in Malawi can be accounted for by climate variations is investigated, while accounting for both observed and unobserved non-climatic confounding factors, spatial heterogeneity and correlation. First, a maximal 'fixed effects’ model was fitted within a negative binomial generalized linear model (GLM) framework [53], to assess the relation between potential predictors and age-stratified counts of malaria cases per month from July 2004 - June 2011. The initial model included the climatic, demographic, socio-economic and cartographic variables described above, with relevant lags and polynomial terms. Categorical variables, to account for the annual cycle, administrative regions and ecological zones, with associated interaction terms, were also tested. With the assistance of a stepwise model selection procedure based on the Akaike Information Criterion (AIC), the model was simplified by removing non-significant interaction terms, quadratic terms and explanatory variables.

Although the GLM accounted for extra variation by the inclusion of climate and non-climate variables and factors, such as the annual cycle and ecological zones, there was still a large proportion of the variance that was unexplained. Consequently, a generalized linear mixed model (GLMM) [54] was adopted. The GLMM is an extension of the GLM that allows for additional variation in the response arising from unobservable random effects. The inclusion of random effects introduces an extra source of variability (a latent effect) into the model to capture the impact of unknown/unobserved confounding factors, such as variations in health care provision, unequally distributed aid or variations in population immunity. Spatially unstructured random effects can assist in modelling overdispersion, previously allowed for solely via the single scale parameter in the negative binomial GLM, while spatially structured random effects allow for correlated heterogeneity between districts. Parameters in a GLMM can be estimated using a Bayesian framework, where parameter uncertainty is accounted for by assigning prior distributions to the parameters. Hierarchical models can be created by parameterizing prior distributions with unknown 'hyperparameters’ which have their own 'hyperprior’ distribution. Markov Chain Monte Carlo (MCMC methods) make estimation of parameters in Bayesian models a practical feasibility [55-57]. This is because associated MCMC sampling yields samples from full posterior predictive distributions, which automatically incorporate all components of variance at the different levels in the model and therefore, provide a full assessment of prediction uncertainty.

When assessing complex Bayesian models, it can be useful to use posterior predictive distributions as reference distributions for comparison to observed data [58]. The posterior predictive distribution of the response is obtained by simulating new pseudo-observations using samples from the posterior distribution of the parameters in the model. The distribution of estimated values can then be compared to observed values. This approach is an alternative to cross-validation, where the model is fitted with part of the data and the remaining observations are compared to the posterior predictive distribution calculated from the sample used for fitting.

### Model formulation

Let *y*_*jst*_ be counts of malaria cases in each age group (*j* = 1,2, where age group 1 represents five years and over and age group 2 represents under five years), district (*s* = 1,…,27) and month (*t* = 1,…,84). After conducting preliminary tests to assess the presence of overdisperion in the count data [59], it was assumed that *y*_*jst*_ arises from a negative binomial distribution

$$\begin{array}{ll} \;y_{\text{jst}}|\mu_{\text{jst}} & ∼\text{NegBin}(\mu_{\text{jst}},\kappa )\\ \;\log\;\mu_{\text{jst}} & =\log\;e_{\text{jst}}+\log\;\rho_{\text{jst}}\\ \;\rho_{\text{jst}} & =\prod_{i=1}^{p}\exp(\theta_{i}x_{\text{ijst}}), \end{array}$$

where *μ*_*jst*_ is the corresponding distribution mean, which is equal to the expected number of cases *e*_*jst*_ multiplied by the unknown relative malaria risk *ρ*_*jst*_ for a given age group *j*, district *s* and time *t*. *κ* is the scale (or overdispersion) parameter and *θ* represents the parameters associated with fixed and random effects included in the model parametrization. Note that population effects are accounted for by including the expected number of cases *e*_*jst*_ (i.e. the population within each district, multiplied by the overall malaria risk) as an offset (see [41] for more details). The model equation can then be rearranged such that the relative risk *ρ*_*jst*_ is equivalent to the standardized morbidity ratio (SMR), where SMR = *y*_*jst*_/*e*_*jst*_, i.e. the ratio of observed to expected cases within a district at a given time. Then, the most suitable estimate of the relative risk *ρ*_*jst*_ (or SMR) is sought via a linear combination of climate covariates (temperature and precipitation) and non-climate confounding factors, both observed, i.e. cartographic, demographic and socio-economic covariates, or unobserved (using random effects) that might explain variations in malaria risk.

Initially for the model selection stage 'fixed effects’, both continuous and categorical, were included in the log-linear predictor $\text{log}\rho_{\text{jst}}=\alpha +\sum_{i}\beta_{i}x_{\text{ijst}}+\sum_{i}\gamma_{i}z_{\text{ijst}}$, where *α* is the model intercept, *β* is the parameter associated with climate covariates *x*_*ijst*_ and *γ* with 'non-climate’ covariates *z*_*ijst*_. Next, random effects were included (hence mixed effects model). As the model parameters were estimated in a Bayesian model framework, prior distributions were specified for all parameters.

Area-specific random effects that are divided into spatially unstructured *ϕ*_*s*_ and structured *υ*_*s*_ components are often termed the 'convolution prior’, *ϕ*_*s*_ + *υ*_*s*_[60,61]. Spatial heterogeneity was introduced by assigning exchangeable location specific random effects using a Gaussian distribution with zero mean and large variance for the unstructured prior $\phi_{s}∼N(0,\sigma_{\phi }^{2})$. Spatial clustering and correlation were accounted for by assigning a conditional intrinsic Gaussian autoregressive model (CAR) to the spatially structured prior, which takes the neighbourhood structure of the districts into account, $\upsilon_{s}∼\text{CAR}(\sigma_{\upsilon }^{2})$[62]. Note that Likoma island, located in Lake Malawi, was excluded from the analysis to facilitate the creation of the neighbourhood structure (hence 27 districts were modelled rather than 28).

Autocorrelated random effects for each calendar month were included to account for the annual cycle of malaria. Since only part of the malaria annual cycle may be attributable to climatic conditions, the inclusion of this effect allowed the model to account for other potential seasonal confounding variables, such as seasonal population movements [63]. Thus, climate variables are retained in the GLMM only if they add additional information that improves the fit of the model. This seasonal term is included as a structured first order autoregressive month effect to account for temporal serial correlation in malaria transmission (e.g., malaria relative risk in one month may depend on the risk in the previous month). The month effect was assigned a random walk or first difference prior distribution, in which each effect is derived from the immediately preceding effect, $\omega_{1(t)}=0,\;\omega_{t^{\prime }(t)}∼N(\omega_{t^{\prime }(t)-1},\sigma_{\omega }^{2}),\;t^{\prime }(t)=2,\ldots ,12$[55]. To account for the apparent trend in the data and unobserved confounding factors, an exchangeable unstructured prior was assigned to the year effect with year 1 (July 2004 - June 2005) set to zero and subsequent years assigned a Gaussian distribution with zero mean and large variance $\tau_{1(t)}=0,\;\tau_{t^{\prime }(t)}∼N(0,\sigma_{\tau }^{2}),\;t^{\prime }(t)=2,\ldots ,7$[61].

Diffuse gamma hyperpriors were assigned to the precisions (1/*σ*^2^) for the spatial and temporal random effects. The specification for the relative risk is then

$$\log\;\rho_{\text{jst}}=\alpha +\underset{i}{\sum }\beta_{i}x_{\text{ijst}}+\underset{i}{\sum }\gamma_{i}z_{\text{ijst}}+\phi_{s}+\upsilon_{s}+\omega_{t^{\prime }(t)}+\tau_{t^{\prime }(t)}.$$

MCMC simulation was used to produce samples of model parameter values from their joint posterior distribution. Two parallel MCMC chains were generated, each of length 25,000 with a burn-in of 20,000 and thinning of 10 to obtain 1000 samples from the joint posterior distribution (see [43]). Convergence was assessed by inspecting plots of traces of simulations for individual parameters and monitoring the Gelman-Rubin diagnostic [64]. Finally, posterior predictive distributions were generated to compare model predictions to observations.

## Results & discussion

### Potential drivers

**Figure 1:**
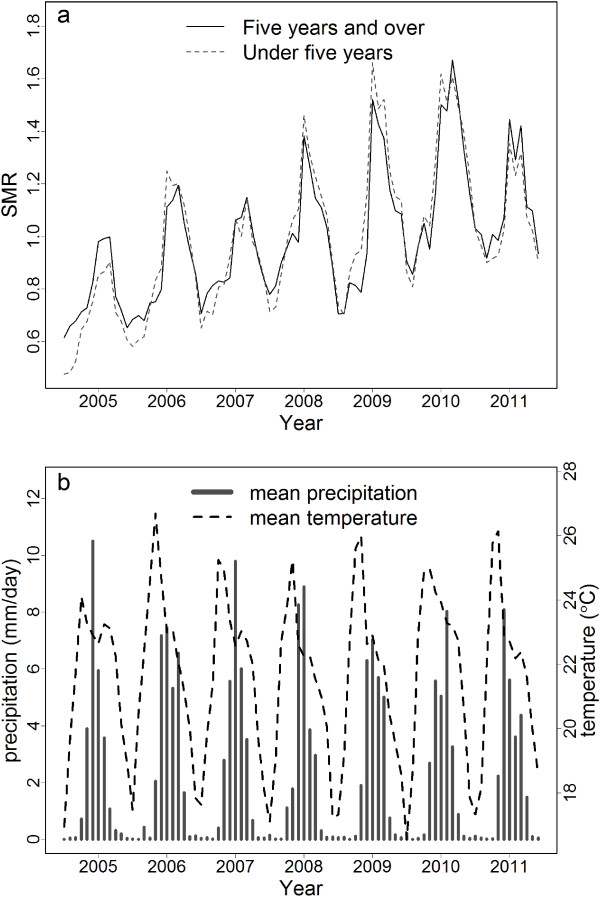
**Malaria SMR and average climate in Malawi for the period July 2004 - June 2011.** **(a)** Malaria standardised morbidity ratios (SMR) for the under five (dashed curve) and five years and over (solid curve) age categories and **(b)** average precipitation (solid bars) and average temperature (dashed curve) in Malawi for the period July 2004 - June 2011.

**Figure 2:**
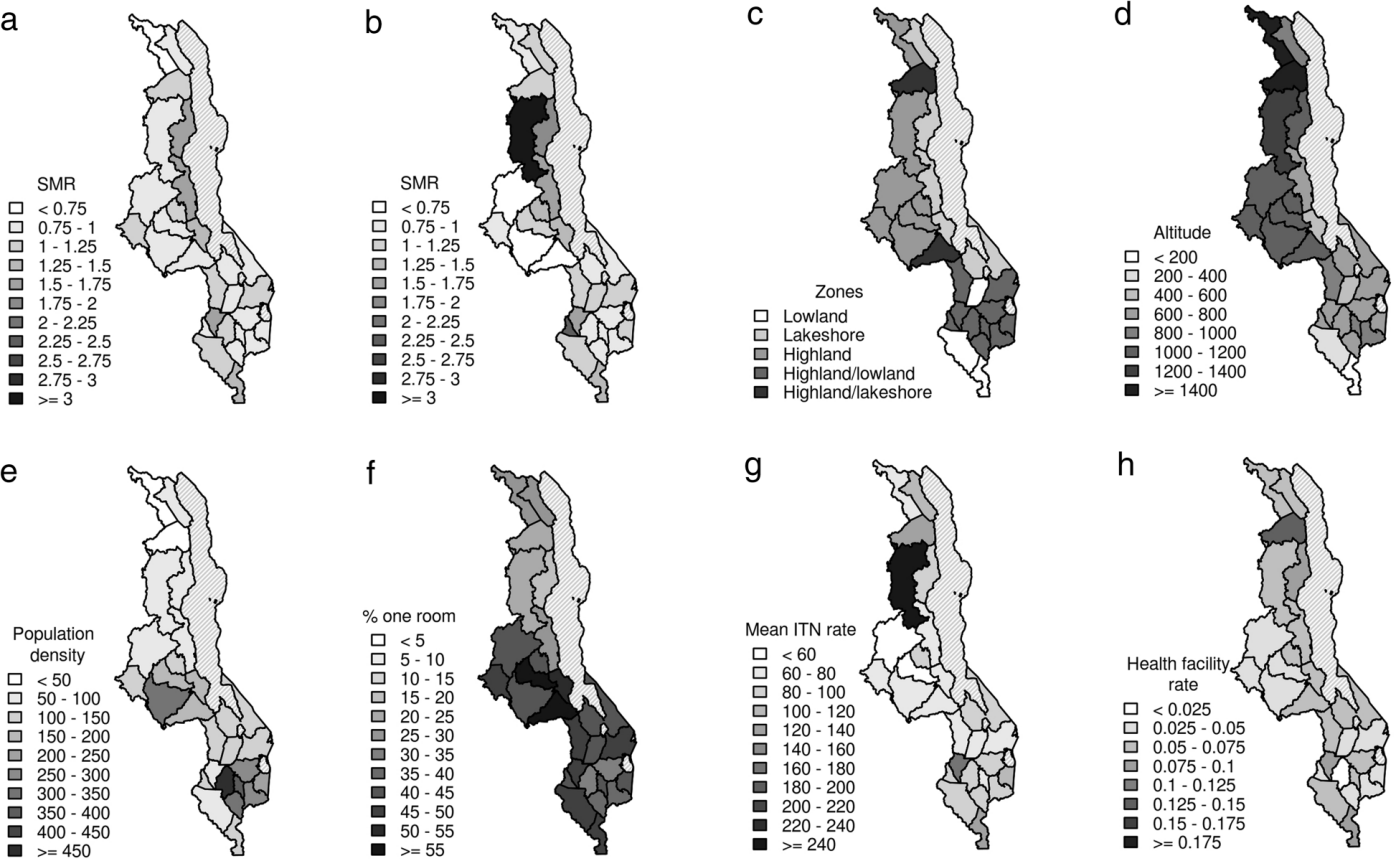
**Spatial distribution of malaria SMR, geographic and socio-economic indicators across Malawi for the period July 2004 - June 2011.** Map of **(a)** malaria SMR for under fives, **(b)** malaria SMR for five years and over, **(c)** ecological zones, **(d)** mean altitude, **(e)** population density, **(f)** proportion of households with only one room for sleeping, **(g)** mean ITN distribution rate and **(h)** the number of health facilities per 1000 inhabitants in each district over the period July 2004 - June 2011.

A precursory view of the temporal and spatial variation of the malaria SMR in Malawi is given in Figure 1 and 2, along with potential driver variables. Figure 1a shows the temporal series of malaria SMR from July 2004 - June 2011 for the under five year and five year and over age categories. A strong annual cycle is apparent, with the peak in the early months of the year. Figure 1b shows the corresponding monthly average precipitation and temperature. The known lag between the malaria transmission season and the rains is clearly apparent. The inter-annual variability in the peak SMR is superimposed on an upward trend over the period. While changes in climate and environmental conditions cannot be ruled out, it is far more likely that this trend is a result of the improved levels of reporting that resulted as districts moved to and became familiar with the electronic based reported system that was introduced in 2004. Figure 2a and 2b show the overall malaria SMR (for under fives and five years and over respectively) in each district over the whole time period (84 months). Figure 2c-f shows the ecological zones, mean altitude, population density, proportion of households with one room for sleeping, the mean ITN distribution rate over the seven year period and the number of health facilities per 1000 inhabitants, respectively.

It is interesting to note that the spatial distribution of SMR for the under fives category broadly reflects the map of prevalence produced by the Malaria Atlas Project (MAP) Bayesian analysis of survey data [65], with higher SMR rates along the western shoreline of lake Malawi and central-west lowlands of the southern part of the country. The adult distribution of SMR reflects the same pattern to a certain extent, with the exception of the high SMR in Mzimba district which was due to an outbreak in April 2006. The greatest contrast to the MAP data is that significant cases are still reported in the northern districts of Chitipa, Rumphi and Karonga, for which the MAP analysis reports very low prevalence rates, although it should be recalled that the MAP survey data is relatively sparse in Chitipa and Karonga districts. In the southern most district of Malawi, Nsanje, a relatively high SMR is observed along with a high proportion of household with only one room for sleeping and also low altitude. Note that the health facility rate is also higher than in surrounding districts.

### Fixed effects model

Using the negative binomial GLM framework specified above (i.e. with only fixed effects), exploratory analyses were conducted to find the best time lags between climate variables and malaria. At the 0.05 level of significance, precipitation and temperature covariates lag 1-3 were found to be statistically significant. Rather than selecting a particular lag, or including all three lags separately, which could result in over-fitting, these variables were combined into three month average precipitation and temperature variables, lagged two months previous to the malaria month of interest. Quadratic terms related to these climate covariates were also tested in order to capture possible non-linear effects, along with various interaction terms. Other geographic and socio-economic variables (listed in Table 1) were tested.

As there may be other spatially varying determinants of disease transmission risk, such as soil type, land cover and land use, for which data was not available, a low order varying proxy location parameter was incorporated in the GLM analysis by including polynomial functions of longitude and latitude, thereby treating location as a continuous predictor. Several categorical variables were also tested including age group (under five years and five years and over) to account for different vulnerability for children under the age of five; calendar month, to model the annual cycle and avoid over-estimating relationships between malaria and climatic variables due to seasonality; year, to account for the trend in the data; region (north, central and south) to account for possible inequalities between the management of malaria between these administratively defined regions; and ecological zone (lakeshore, lowland, highland, highland/lakeshore, highland/lowland). This preliminary analysis was assisted by use of a model selection algorithm based on the AIC stepwise regression [53]. In order to compare the fixed effects model with subsequent models including random effects, model parameters were estimated within a Bayesian framework using MCMC and were considered to be statistically significant if their 95% credible interval did not contain zero. Note that all continuous variables were first standardized to zero mean and unit variance to aid MCMC convergence. Table 2 shows the parameter estimates and 95% credible interval for the continuous explanatory variables that were retained in the model as statistically significant, and resulted in the lowest deviance information criterion (DIC) [66] (indicating goodness of fit). Certain variables, such as the proportion of households with no toilet facilities, the proportion of the adult population who never attended school and literacy rates were not found to be statistically significant and did not improve the fit of the model. Note that the categorical variables age, month, year, region and ecological zone were also found to be statistically significant.

**Table 2:** Parameter estimates for statistically significant continuous explanatory variables for selected fixed effects model (GLM)

**Variable**	**Parameter**	**95% credible**
	**estimate**	**interval**
Altitude	-0.263	(-0.338, -0.192)
Longitude	1.141	(0.321, 2.256)
Longitude^2^	-1.285	(-2.388, -0.469)
Latitude	-3.108	(-3.425, -2.840)
Latitude^2^	-2.935	(-3.315, -2.630)
Urban population	-0.039	(-0.062, -0.019)
One room	-0.101	(-0.128, -0.075)
ITN/population	0.049	(0.031, 0.068)
Health facilities/population	0.079	(0.057, 0.100)
Traditional housing	-0.026	(-0.047, -0.002)
Rainfall	0.190	(0.134, 0.239)
Rainfall^2^	-0.063	(-0.085, -0.040)
Temperature	0.073	(0.030, 0.127)
Temperature^2^	-0.014	(-0.027, -0.001)

### Mixed effects model

As standard statistical regression models assume independent (or at least uncorrelated) observations, a mixed effects model (GLMM) was fitted in a Bayesian hierarchical modelling framework to account for spatial and temporal dependence. The continuous and categorical variables selected in the fixed effects model were included. However, a random walk was introduced in the annual cycle, to account for dependencies between one month to the next and exchangeable random year effects were assigned to account for the trend and unobserved confounding factors. Spatial heterogeneity and correlation were accounted for using the convolution prior, described in the Model formulation section. Interestingly, all the previously selected variables, except average temperature, rainfall (quadratic relation), age group, the ITN distribution rate and proportion of health facilities per inhabitant, ceased to be statistically significant (i.e. the credible interval contained zero).

**Figure 3:**
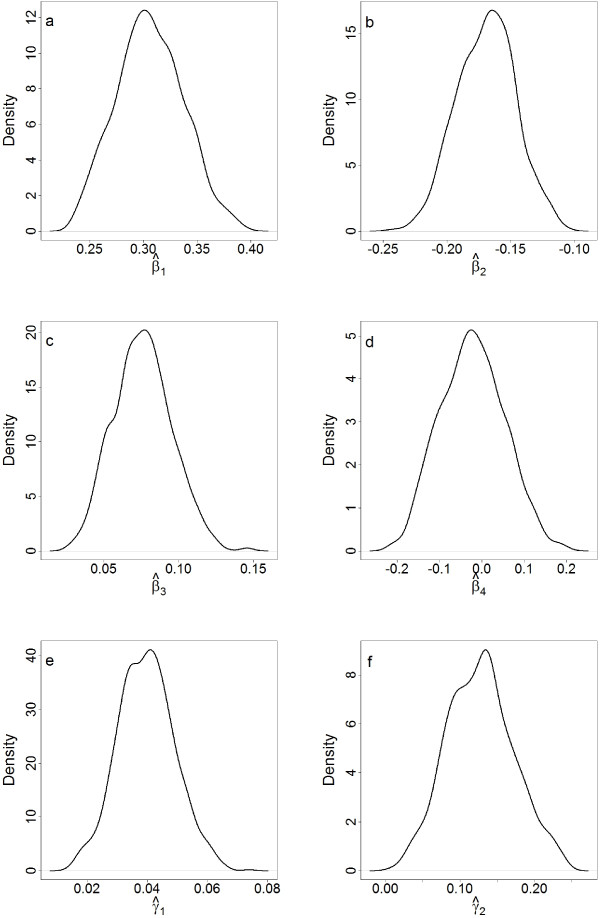
**Kernel density estimates for significant explanatory variables.** Kernel density estimates for the marginal posterior distributions for the parameters associated with **(a)** average precipitation, **(b)** precipitation squared, **(c)** average temperature **(d)** temperature squared, **(e)** health facilities per inhabitant and **(f)** ITN distribution rate.

Figure 3 shows the kernel density estimates for the marginal posterior distributions for the statistically significant parameters associated with the variables rainfall and rainfall squared, temperature and temperature squared, the proportion of health facilities per inhabitant and the ITN distribution rate. As in the fixed effects model, a statistically significant quadratic relation between average rainfall during the proceeding three months and malaria risk was found (see Figures 3a and 3b). Although temperature was statistically significant, once confounding factors were accounted for, a quadratic relationship between temperature and malaria risk in Malawi was not found to be statistically significant (see Figure 3d). This is likely due to the monthly average temperature range (15.8 - 28.9°C) in Malawi over the time period not exceeding values at which mosquito activity is suppressed [13].

As in the fixed effects model, the number of health facilities per inhabitant was positively associated to malaria relative risk, as was the ITN distribution rate (see Figures 3e and 3f). At first this appears contrary to expectations, since improved access to medical treatment and preventative measures is expected to reduce the parasite burden in the population and hence, the transmission intensity, resulting in an inverse relationship. The positive relationship observed most likely reflects more frequent reporting and greater distribution of ITNs where more health facilities are present, along with possible targeting of ITN distribution by donors in highly burdened districts. This result potentially highlights the gap between ITN possession and proper use [67]. According to Amexo *et al.*[68] 70% of people in Africa self-diagnose malaria and self-treat at home. It is likely that the proportion of the population reporting promptly increases with proximity to health centres. The construction of new health centres is determined by the distance that members of a community have to walk to an already existing facility. The Government of Malawi recommends that the population should live within an 8km radius of a health facility. New health centres are constructed in order to attain this target. In a study conducted by the Ministry of Health, some districts were found to be better served than others. For example, 51% of the population in sparsely populated Chitipa district lives more 8km from a health facility. In Blantyre, Chiradzulu, Mulanje and Zomba less than 5% of the population reside more than 8km from a health facility [69].

**Figure 4:**
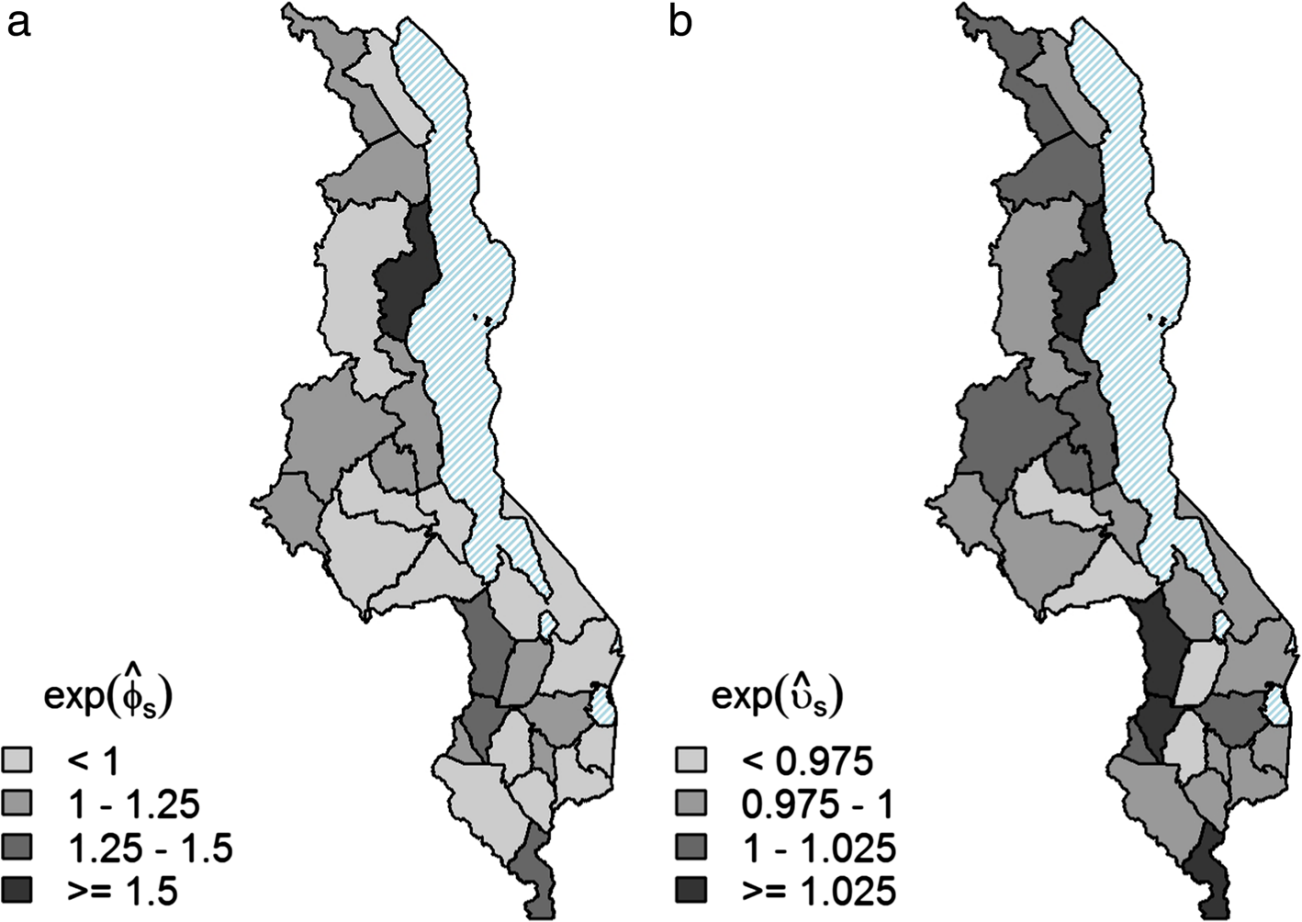
**Multiplicative contribution of spatially unstructured and structured random effects to malaria relative risk.** Spatial distribution of multiplicative contribution of posterior mean spatially **(a)** unstructured ${\hat{\phi }}_{s}$ and **(b)** structured ${\hat{\upsilon }}_{s}$ random effects.

**Figure 5:**
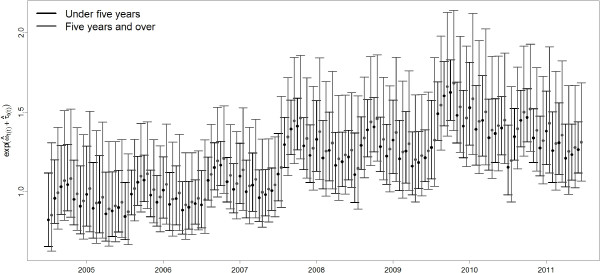
**Multiplicative contribution of temporally unstructured and structured random effects to malaria relative risk.** Temporal distribution of auto-correlated random month effects ${\hat{\omega }}_{t(t)}$ (accounting for annual cycle) and random year effects *τ*_*t*(*t*)_ (to account for unexplained trend) for under five and five years and over age categories.

Figure 4 shows the multiplicative effect of the two components of the convolution prior to the model. The key feature of the convolution prior is that it allows the assessment of relative contributions of unstructured heterogeneity and spatial clustering to the overall variation of the area effects [70]. From Figure 4 it is evident that spatial heterogeneity is the dominant cause of overdispersion in Malawi. The spatially unstructured random effect *ϕ*_*s*_ accounts for residual overdispersion in districts that is not attributable to spatial correlation between districts. Here, the spatial correlation component has a minimal yet significant contribution to the convolution prior. Although other geographic and socio-economic covariates such as altitude, longitude, latitude, ecological zone, region, and proportion of the population in each district residing in traditional housing were significant in the fixed effects model, they became non-significant in the mixed effects model. This demonstrates the importance of accounting for spatial heterogeneity and correlation, when analysing geographical data in order to avoid under-estimation of the credible intervals of model covariates. The structure of the random spatial component of the model provides a combined measure of the various potential risk factors that might contribute to the underlying spatial variation in malaria risk. The advantage is that only two hyperpriors are estimated for the precisions of spatial random effects, rather than numerous parameters for each different fixed effect. This results in a more parsimonious model, containing few strong predictors that are more easily interpretable. Figure 5 shows the contribution of the auto-correlated annual cycle and random yearly effects to the malaria relative risk, stratified by age group (under five years and five years and over) over the period July 2004 to June 2011. These effects help account for the annual cycle in malaria, that could be attributed to climate and/or seasonal population movements, and the overall upward trend that could be the result of improved reporting over the years as the health facilities became accustomed to the newly established HMIS.

### The role of climate in estimating malaria relative risk

**Figure 6:**
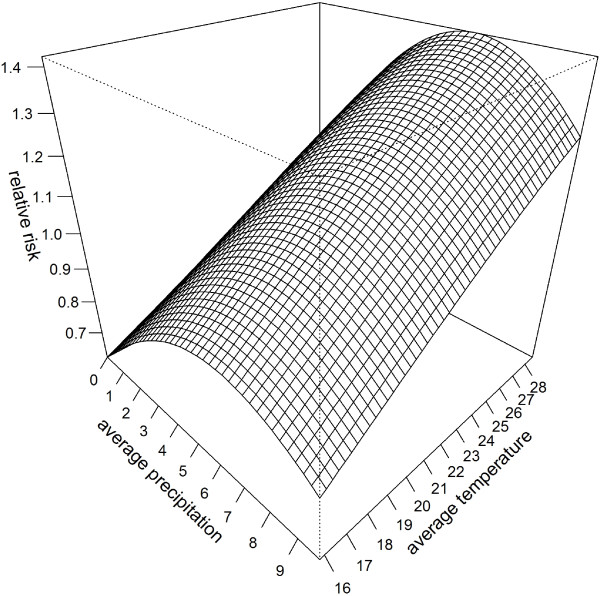
**Multiplicative contribution of climate variables to malaria relative risk.** Surface of malaria relative risk given varying average precipitation and temperature values. Note that the maximum relative risk is found at the maximum temperature of 28°C and a precipitation threshold of 6.24 mm day^-1^.

Figure 6 shows a surface of the multiplicative contribution of climate variables to malaria relative risk in Malawi. Given varying average precipitation and temperature values, the maximum relative risk is found at the maximum temperature of 28°C and a precipitation rate of 6.24 mm day^-1^. This result is supported by other studies, for example, a quadratic relationship between malaria incidence and rainfall was found in Botswana [20]. This likely relates to the wash out of first stage larvae from breeding sites by intense rainfall. These effects have been included in some dynamical models of malaria transmission either implicitly [71] or explicitly [31].

**Figure 7:**
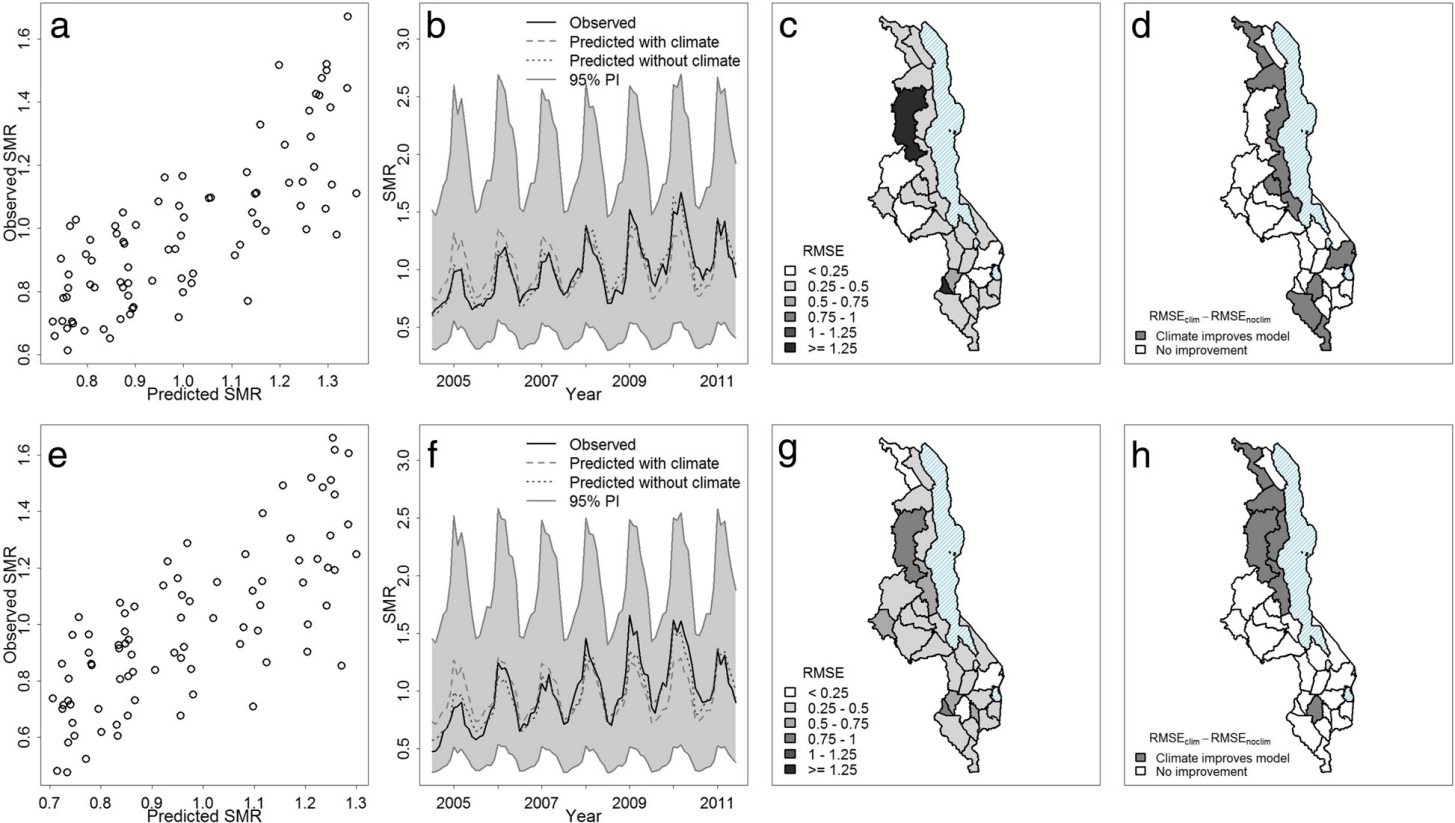
**Observed versus predicted malaria SMR in space and time.** Scatter plot **(a, e)** and time series **(b, f)** of space aggregated observed versus posterior predictive mean malaria SMR for the 84 month time period. Root mean squared error (RMSE) of observed and posterior mean malaria SMR for the 27 districts of Malawi for the period July 2004 - June 2011 **(c, g)** for the five years and over (upper panel) and under five years (lower panel) age groups. The lower the RMSE, the better the model fit. Difference between RMSE for the model including climate information and RMSE for a model fit without climate information **(d, h)**. Districts with negative values of RMSE_*clim*_ - RMSE_*noclim*_ (white) suggest that climate information improves the model in these areas. Districts with positive values of RMSE_*clim*_ - RMSE_*noclim*_ (grey) suggest that climate information does not improve the model.

To assess the predictive ability of the mixed effects model, posterior predictive distributions of malaria relative risk were obtained for each district and month. New pseudo-observations were simulated by drawing random values from a negative binomial distribution with mean and scale parameter estimated using samples from the posterior distribution of the parameters in the model. To summarize this information, the observed and posterior predictive mean malaria risk (SMR) estimates were aggregated across space. Figure 7 shows scatter plots and time series of observed versus predicted malaria SMR for the 84 month period for age groups five years and over (upper panel) and under five years (lower panel). To evaluate the model across space, the root mean squared error (RMSE), a measure of the difference between model predicted and observed values, was calculated over the 84 month period for each district. For both age groups there is an overall positive agreement between predicted and observed space aggregated malaria risk (Figures 7a and 7e). Both time series plots for the two age groups averaged over the whole of Malawi show that the model is able to capture the inter-seasonal variability. In order to assess how much additional inter-seasonal and inter-annual variability is explained by the climate covariates, climate was removed from the model and a prediction without climate was superimposed on the plot (Figures 7b and 7f). In general, when averaging across the country, little or no improvement in malaria relative risk estimation is achieved by the addition of climate covariates in the model. Figure 7 (c and g) show maps comparing observed to predicted malaria risk in each district. Relatively low values of RMSE are found, particularly in the southern districts of Malawi. In general, crude maps of SMRs are subject to considerable random error, particularly if the population count within a district is low. Therefore, visual attention is drawn to areas where rates are based on the least stable estimates, for example, the large but sparsely population Mzimba district (north region). The inclusion of random effects in the model framework makes it possible to 'borrow strength’ from neighbouring districts, resulting in spatial smoothing of risk surfaces. Therefore, the greater difference between observed and predicted malaria risk for Mzimba may in fact highlight the poor reliability of the data in this area.

To assess whether the inclusion of climate information in the model could improve model estimation of malaria relative risk at the district level from year to year, the RMSE of the model excluding climate covariates, RMSE_*noclim*_, was subtracted from the RMSE of the model including climate covariates, RMSE_*clim*_. Areas where RMSE_*clim*_ - RMSE_*noclim*_ < 0, highlighted in grey in Figure 7 (d and h), indicate that climate information improves the estimation of malaria relative risk, as the inclusion of these covariates results in a smaller difference between the model predicted values and the observations. Therefore, according to this model, climate information could help estimate malaria relative risk in under fives and five years and over several months ahead in the districts: Blantyre, Chikhwawa, Chitipa, Machinga, Mzimba, Nkhata Bay, Nkhotakota, Nsanje, Ntchisi, Rumphi and Salima. This represents 41% of the districts in Malawi.

It is interesting to note that many of the districts, where climate is found to improve the model, are located in the north of the country. In this region, away from the lake-side communities, prevalence is generally much lower, due to the altitude and lower temperatures [65]. In these circumstances, inter-annual variability in climate can intermittently lead to years with wetter or warmer conditions, which can result in more intense malaria transmission. In contrast, in the low-lying southern districts, where climate is conducive to intense transmission, year to year climate perturbations are perhaps not expected to impact morbidity. Instead, changes to socio-economic conditions and interventions may dominate. In addition, it should be recalled that the spatial resolution of the district scale data may average out climatic effects if districts include widely varying terrain.

Although the model is able to identify the relative importance of climatic, geographic and socio-economic determinants of malaria in Malawi, this study has several limitations. Firstly, malaria data was only available for the whole of Malawi at the relatively coarse spatial resolution of the district-level. One advantage of using aggregated data is to alleviate problems of misreporting due to variations in the diagnostic capabilities and reporting practices between individual health facilities. However, given this limitation and the coarse resolution of the climate data, the model formulated is unable to capture sub-district variations in malaria, which are likely influenced by localized meteorological and social conditions.

Secondly, the statistical analysis is limited by the short time period for which malaria data is available. Nevertheless, the information gained from the spatial component allows some inference to be drawn as the role of climate and other factors in the transmission of malaria in Malawi which can also be used for improvement and evaluation of dynamical transmission model [31,72]. As time goes by and the established monitoring system provides a clearer long-term picture of malaria transmission, more detailed temporal and spatial information can be included in the model.

Thirdly, malaria data was obtained from case records at clinics, health facilities and hospitals. Therefore, malaria cases treated at home are missed. As malaria is endemic in much of the country, data incurs the omission of asymptomatic cases. Both home treated and asymptomatic cases result in underestimation. However, overestimation can occur when health facilities report suspected cases of malaria that are not clinically confirmed. In fact, it is estimated that only 37% of fever cases in children in Malawi are actually due to malaria [73]. Although there is no specific measure included in the model to account for misreporting over time, the health care provision indicator gives a broad indication of the accuracy of recording, while the spatial and temporal random effects account for potential unmeasured variability.

This work compliments the spatial risk maps of malaria that have been produced using finer scale point-referenced prevalence of infection data for Malawi [37]. An advantage of using point referenced data obtained from surveys such as the malaria indicator survey (MIS) is that malaria in children under five years of age is clinically diagnosed using RDTs, thus preventing over estimation of cases. The MIS for Malawi was first conducted in 2010 and will be repeated every two years, thus allowing the incorporation of temporal effects in such models. By combining geostatistical and process-based modelling approaches, spatio-temporal predictions of malaria risk may be possible at a finer spatial scale in areas where data is not recorded. However, there is an on-going need for continued collaboration between statisticians, mathematical modellers, meteorologists, public health decision makers and stakeholders in Malawi to construct models and interpret model results. Despite the limitations of this case study, due to the relatively coarse spatial resolution, short time series and data quality issues, with careful model selection this sophisticated modelling framework could serve as a useful tool to understand the relationship between climate, geographic and socio-economic conditions and malaria burden in other countries.

## Conclusion

The main contribution of this paper is the collation of a unique dataset of potential spatial and temporal drivers of malaria in Malawi and the use of a sophisticated modelling procedure to determine the most important of these drivers. An initial model was selected that contained statistically significant fixed effects. After accounting for spatial heterogeneity and correlation, the mixed effects model was reduced to contain a few predictors that are easily interpretable, including average temperature and rainfall. Including climate information improves the estimation of inter-annual variations in malaria relative risk in 41% of the districts in Malawi, some of which are located in the north highland regions that are subject to lower and intermittent malaria transmission intensity (with the exception of lake-side communities). In the southern region, where malaria transmission is more intense, climate improved the model’s capability to represent year to year variations in malaria relative risk in only a few districts.

While this analysis has the common caveats associated with reliability and limited time-span of health data, this is the first spatio-temporal model for malaria relative risk in Malawi, at the district level. The analysis indicates that a climate-based early warning system could have some value in Malawi’s northern epidemic-prone districts and emphasizes the critical requirement for an effective climate monitoring system, and access to high quality climate forecasts. A climate-based malaria decision support system could be invaluable for the Malawi National Malaria Control Programme to be able to annually plan their locally targeted control interventions and manage scarce health resources.

## Abbreviations

AIC: Akaike Information Criterion; CAR: Conditional autoregressive (model); DIC: Deviance information criterion; ECMWF: European Centre for Medium Range Weather Forecasts; GLM: Generalized linear model; GLMM: Generalized linear mixed model; HMIS: Health Management Information Systems; ITN: Insecticide treated net; MAP: Malaria Atlas Project; MCMC: Markov Chain Monte Carlo; MIS: Malaria indicator survey; MOH: Ministry of Health; NGOs: Non Governmental Organizations; NSO: National Statistical Office; RDT: Rapid diagnostic tests; RSME: Root mean square error; SMR: Standardized morbidity ratio; WHO: World Health Organization; WMS: Welfare Monitoring Survey.

## Competing interests

The authors declare that they have no competing interests.

## Authors’ contributions

RL collated the data, devised the model framework and wrote the manuscript. JC provided data and local expert advice, helped collate the data and contributed to the manuscript. AMT helped compile the climatic data, design the analysis and contributed to the manuscript. All authors read and approved the final manuscript.
